# Assessment of manual operation note documentation practice: a cross-sectional study

**DOI:** 10.1097/MS9.0000000000001124

**Published:** 2023-08-07

**Authors:** Nega Getachew Tegegne, Demeke Yilkal Fentie, Biresaw Ayen Tegegne, Belete Muluadam Admassie

**Affiliations:** a Department of Anesthesia, College of Medicine and Health Sciences, Debre Tabor University, South Gondar; b Department of Anesthesia, College of Medicine and Health Sciences, University of Gondar, North Gondar, Ethiopia

**Keywords:** documentation, medical records, operation notes, surgical note

## Abstract

**Background:**

Operation note documentation captures the key findings and subtle elements of a surgical strategy and is crucial for patient safety. Poor operation note documentation can negatively influence postoperative patient care. This study aimed to assess manual operation note documentation practice.

**Methods:**

An institutional-based, cross-sectional study was conducted from 30 March to 30 April 2022, on 240 operation notes of patient data. Data were entered and analyzed by SPSS version 20. According to the RCSE, the Royal College of Surgeons of England, the practice of operation note documentation was rated excellent for each variable when it met 100%, good if it met more than 50%, and poor if it met less than 50% of the operation notes of patient data.

**Results:**

All operation notes (*n*=240) were handwritten. The practice of manual operation note documentation was deemed excellent in two (7.69%), good in 18 (69.2%), and poor in six (23.1%). Residents wrote 84.2% of the operation notes and surgeons and assistants were identified in greater than 94% of the notes, while anesthesia team members were identified in 90.8%. Estimated blood loss was documented in 4.2% of the notes, and the closure technique was described in 64.2%. The operation note templates did not include antibiotic prophylaxis, runner nurse name, or gauze and instrument counts. The urgency of the surgery and time of documentation had a negative relationship, and the seniority of the operation note writer had a positive relationship with manual operative note documentation practice.

**Conclusions and recommendations:**

Compared to the standard, all operation note documentation was incomplete and below the standard. We recommend that this comprehensive and specialized hospital administrator implement a new format for operation notes that incorporates RCSE requirements.

## Introduction

Highlights

- All operation notes were handwritten.
- Most of the operation note was incomplete and did not adhere to the standard.
- Adherence to the RCSE (Regal College of Surgeons of England) criteria of 7.69% was deemed excellent.

Operation notes documentation captures the details of a surgical procedure, counting individual details of the patient, indications for surgery, technical descriptions, intraoperative findings, and postoperative instructions^1^. Clinical record keeping is an essential component of providing quality healthcare^2^.

Both from a therapeutic and legal point of view, the quality of operative notes is vital to improving communication between distinctive healthcare experts^3^. Because operative notes are frequently presented in legal malpractice cases, studies have shown that up to 45% of operative notes are medico-legally indefensible^4^.

Evidence from the National Confidential Enquiry into Patient Outcome and Death (NCEPOD) reveals that poor documentation can negatively influence postoperative care at several stages, for example immediately postoperatively, predischarge, and subsequent patient care^5^.

The General Medical Council recommends that accurate, comprehensive, and legible records be maintained for every patient by the surgeon^6^. Accurate, legible, and detailed operation notes are of great importance in all surgical specialties, not only for patient care but also for providing information for research, audit, postoperative management, and medico-legal purposes^7^. Handwritten notes are still used worldwide; however, establishing their legibility can often be a major drawback^8^.

An audit conducted in Kuwait by Sweed *et al*.^9^ illustrated that 20% of the operative notes they looked at contained illegible parts, were incomplete, and included confusing abbreviations. Audits of operation quality regularly identify failure to meet the required standards^3,10–13^.

In 2009, the National Patient Safety Agency (NPSA) looked in detail at the causative factors in patient safety incidents (PSIs)^14^. Human factors were a major contributor to PSIs, and, in particular, communication issues were highlighted. Written communication plays a critical role in all perspectives of mistakes, with illegible handwriting and vague instructions highlighted as common issues^15^.

To improve our clinical practice, there is a need to adopt a standardized way to record operative notes so that our records contain all the details fundamental to delivering patients the best possible care. Although there are limited standardized guidelines in Ethiopia relating to operative notes, there are worldwide guidelines that are in use and are well-perceived, such as those set by the Royal College of Surgeons of England (RCSE)^13^.

The Good Surgical Practice guidelines published by the RCSE recommend that operation notes be legible, typed (if possible), and taken with the patient from the working operation theater to recovery and the surgical ward^1^.

Royal College of Surgeon guidelines have mainly identified criteria for operation note documentation for ‘good surgical practice’^13^.

Evidence from the NCEPOD reveals that poor documentation can negatively influence postoperative care at several stages, for example immediately postoperatively, predischarge, and subsequent long-term patient care^5^.

As a result, accurate and clear notes benefit the patient by serving as a tool for communication between anesthesiologists and their medical and nursing colleagues^4^.

A few studies have been conducted on operation note documentation in the study setting. Therefore, this study aimed to assess the adherence of operation note documentation against the standards set by RCSE to improve the quality of operative notes and ensure immediate postoperative care, for subsequent management, in hospital care and also for referred as well as respective surgical, nursing, and anesthetic patient care.

## Methodology

Ethical clearance was obtained from the institutional ethical review committee. The aim of the study was explained to each study participant, and informed consent was obtained. This study was registered with the UIN research registry and reported following STROCSS (strengthening the reporting of cohort, cross-sectional and case–control studies in surgery) criteria^16^.

### Study design

An institutional-based, cross-sectional study.

### Performed setting

This comprehensive and specialized hospital is among the largest hospitals, with over 500 beds, of which ~200 are surgical beds, including trauma, obstetrics and gynecology, and ophthalmology.

This provides inpatient care, outpatient services, and follow-up clinics, covering elective and emergency surgery, with six operating theaters, one orthopedic table in one operation room complex, a minor operating theater, and 24-h emergency service.

In this study, we reviewed the operation notes for patients admitted for surgery from 30 March to 30 April 2022, using the updated 2014 RCSE guidelines as a quality benchmark^1^.

### Source population

All operation notes are documented in the study area.

### Sample population

Operation notes were documented in the study area during the study period.

### Sample size

The required sample size was calculated using a single population proportion formula from a previous study (45%)^17^ as follows:


n=(Z/2)2p(1−p)/d2.

Assumptions are *n* is the required sample size, *Z* is the critical value for normal distribution at a 95% confidence level (1.96), *W*=0.05 (5% margin of error), and *E* is the best estimate of the population proportion.


n=(1.96)2×(0.45×0.55)(0.05)2=380.16=381.

The correction formula was used. Since our sample population (*N*) is less than 10 000 (i.e. on average, 450 surgeries are done per month in the all-operation room in the study area), we use the following formula to calculate the exact sample size:


nixN/ni+N=nf,

nf=381×450/381+450,

nf=206.3=207

was the total sample size.

Finally, a total of 240 randomly selected operation notes were reviewed in this study and analyzed prospectively for completeness and appropriate documentation.

### Variables of the study

#### Dependant variable

The practice of manual operation note documentation.

#### Independent variable

Urgency of the surgery, Seniority of surgeons who wrote operation notes, time of documentation.

### Operational definition

The practice of manual operation note documentation was deemed excellent if met at 100%, good if it met at >50%, and was deemed poor if met at <50% of each variable of patient data^17^.

### Data collection method

The principal investigator and one bachelor of degree Anesthesia student reviewed 240 operation notes from the record written by the operating surgical team over 1 month.

The data were checked, coded, entered, and cleaned using SPSS version 20. A descriptive statistical analysis was performed. Results were expressed in frequency tables, charts, and percentages.

All the data were collected based on the RCSE operation note writing guideline standard format (Tables 1 and 2) and directly changed into question forms with three integral checking components: ‘Yes’, ‘No’, and ‘partial’.

**Table 1:** Sociodemographic variables for the practice of manual operation note documentation

Serial number	Sociodemographic variables	RCSE target (%)	Evidence	Data sources could be
1	Date	100	RCSE	Operation note review
2	Performed operation	100	RCSE	Operation note review
3	Patient’s details	100	RCSE	Operation note review
4	Hospital number	100	RCSE	Operation note review
5	Operating surgeon name	100	RCSE	Operation note review
6	Assistant surgeon name	100	RCSE	Operation note review
7	Anesthetist name	100	RCSE	Operation note review
8	Scrub nurse’s name	100	RCSE	Operation note review
9	Runner nurse’s name	100	RCSE	Operation note review

RCSE, Royal College of Surgeons of England.

**Table 2:** Procedure-related variables for the practice of manual operation note documentation

Serial number	Procedure-related variables	RCSE target (%)	Evidence	Data sources could be
1	Indication	100	RCSE	Operation note review
2	Urgency	100	RCSE	Operation note review
3	Type of anesthesia	100	RCSE	Operation note review
4	Patient position	100	RCSE	Operation note review
5	Type of incision	100	RCSE	Operation note review
6	Operative diagnosis	100	RCSE	Operation note review
7	Intraoperative findings	100	RCSE	Operation note review
8	Problem/complications	100	RCSE	Operation note review
9	Used instrument count	100	RCSE	Operation note review
10	Extra procedure conducted	100	RCSE	Operation note review
11	Removed tissues details	100	RCSE	Operation note review
12	Closure technique	100	RCSE	Operation note review
13	Estimated blood loss	100	RCSE	Operation note review
14	Antibiotic prophylaxis	100	RCSE	Operation note review
15	Postoperative plans	100	RCSE	Operation note review
16	Follow-up plans	100	RCSE	Operation note review
17	Name and signature of writer	100	RCSE	Operation note review

RCSE, Royal College of Surgeons of England.

## Results

From randomly selected and reviewed 240 operation notes and we found that they were not utilized to type the operation notes. Residents wrote about 84.2% of operation notes and consultant surgeons wrote only 15.8% of notes. Most of the operation notes 180 (75%) were documented during the night shift. In our setup during the night shift, emergency surgery was done.

The date of the surgery was documented in almost all of the operation notes reviewed (99.2% of notes); also, the names of the operating surgeon and assistants were consistently documented (above 90% of notes documented the names of healthcare providers in the operating theater). Even though none of the operation notes included the names of runner nurses.

While the primary provider was consistently documented, this was not consistent for other participants. The names of the anesthetists were documented in 90.8% of the notes. Even though the type of anesthesia and the intraoperative findings were documented in more than 95% of the notes, the position of the patient, the type of incision, the operative diagnosis, and the intraoperative findings were documented in less than 75% of the notes (Tables 3 and 4). Also, the occurrence or nonoccurrence of complications was inconsistently documented, being included in 5.9% of the notes.

**Table 3:** The relation of sociodemographic variables with the practice of manual operation note documentation (*n*=240)

Standards	The frequency (%) of operation notes met the standard	The frequency (%) of operation notes did not meet the standard
The operation notes which were dated	238 (99.2)	2 (0.8)
The performed operation was stated	212 (88.3)	28 (11.7)
The hospital number of the patient was documented	240 (100)	–
Indication of the procedure was stated	214 (89.2)	26 (10.8)
The urgency of the procedure was documented	4 (1.7)	236 (98.3)
Surgeon’s name was stated	240 (100)	–
The assistant surgeon’s name was stated	226 (94.2)	14 (5.8)
The anesthetist’s name was stated	218 (90.8)	22 (9.2)
The scrub nurse’s name was stated	224 (93.3)	16 (6.7)
The runner nurse’s name was stated	–	240 (100)

**Table 4:** The relation of procedure-related variables with the practice of manual operation note documentation (*n*=240)

Type of anesthesia was documented	234 (97.5)	6 (2.5)
Position of the patient was documented	178 (74.2)	62 (25.8)
Type of incision was documented	166 (69.2)	74 (30.8)
Operative diagnosis was documented	176 (73.3)	64 (26.7)
Intraoperative findings were documented	232 (96.7)	8 (3.3)
Any problem/complications were documented	14 (5.9)	226 (94.2)
Used instrument count was documented	–	240 (100)
Any extra procedure conducted was documented	24 (10)	216 (90)
Details of the tissues removed were documented	208 (86.7)	32 (13.3)
Anticipated blood loss was documented	10 (4.2)	230 (95.8)
Antibiotic prophylaxis was documented	4 (1.7)	236 (98.3)
Postoperative plans were documented	226 (94.2)	14 (5.8)
Follow-up plan was documented	186 (77.5)	54 (22.5)

The documentation of whether additional procedures were performed other than those initially planned was similarly inconsistent, occurring in 10% of notes. The details of tissue removal and wound closure were documented in 86.7% and 64.2% of notes, respectively. The urgency of the procedure was reported in 1.7% (*n*=240) of the notes, and gauge and instrument counts were not documented in all notes.

Only 7.69% of the RCSE criteria (patient hospital number and operating surgeon name) and adherence to RCSE operation note standards were deemed excellent (100%). However, 23.1% of the RCSE criteria (antibiotic prophylaxis, anticipated blood loss, used instrument count, any problems or complications, the runner nurse’s name, and the procedure’s urgency) did not adhere to RCSE operation note standards (50%).

A postoperative plan was included in nearly all of the operation notes (94.2%). The majority of the operative notes were written by the junior resident (62.5%; *n*=150), followed by the senior resident (21.1%; *n*=54) and the consultant (15.8%; *n*=38). There are also areas that were only partially documented, such as patient detail documentation, wound closure technique, and the identity of the writer of the operation note (Table 5).

**Table 5:** Partially documented variables for practice of manual operation note documentation (*N*=240)

Standards	Frequency (%) of operation notes met the standard	Frequency (%) of operation notes not met the standard	Frequency (%) of operation notes partially met the standard
Patient details were documented	234 (97.5)	–	6 (2.3)
Technique of the closure was documented	154 (64.2)	82 (34.2)	4 (1.7)
Name and signature of the operation note writer were documented	208 (86.7)	2 (0.8)	30 (12.5)

## Discussion

Accurate and complete operative notes are considered a critical element of quality assurance in surgery, and it is among the most important skills required of a surgeon, as appropriate documentation of a surgical procedure is vital for postoperative patient care^18^. The fact that no operation note was appropriately filled out in this study is a reflection of the suboptimal quality of documentation of perioperative details of patients, and this calls for improvement. Audits from Nigeria and other parts of Africa have demonstrated similar, and in some instances, poorer, results^19–21^. Those from developed countries, however, showed marginally better results^22,23^.

Incomplete operation notes hinder postoperative patient management, as notes written with illegible handwriting or those that use nonstandard abbreviations, for example can confuse healthcare providers responsible for further patient care^4^. Even though RCSE guidelines provide an accepted international standard, the operation notes analyzed in this study had not consistently confirmed.

Moreover, incomplete notes are not useful in medico-legal cases, with one study reporting that up to 45% of operation notes cannot be used to support a defendant in a court of law^4^. In this study, the date of the surgery was documented in almost all notes (99.2%), which is similar to findings from other studies in Africa and elsewhere (92.6–99%)^18,20^.

RCSE guidelines stipulate that both date and time should be recorded in operation notes; this was consistently documented at the hospital under study. Contrary to this, the time of surgery was commonly omitted in operation notes evaluated by investigators in Nigeria and Pakistan^24,25^; however, a study conducted in Sudan found that the time was documented in 81% of notes^20^.

In the current study, the names of surgical team members were documented fairly consistently in the notes, but the runner nurses were missed from all of the notes. This may have been due to differences in the formatting of the operation note forms used. A study from Sudan found that the names of the anesthetists and scrub nurses were rarely documented (13.9 and 0.9%, respectively)^20^; in comparison to this, our audit found that the scrub nurses’ and anesthetists’ names were mentioned in above 90% of notes.

This demonstrates an opportunity for a revised operation record sheet format, which, if linked to a preoperative safety checklist, would facilitate the introduction of all surgical team members and the documentation of their names^26^.

In this study, the type of anesthesia and intraoperative findings were documented in more than 90% of the notes that we investigated, and this was comparable with previous studies^26^. Position of the patient, type of incision, operative diagnosis, and closure technique were less frequently documented, which was lower than the rates observed in the previously mentioned studies conducted in Nigeria (82%)^25^ and comparable with the study conducted in Pakistan (69%)^24^, but higher than the proportion observed in the study carried out in Sudan (26.9%)^20^.

According to our findings, the majority of operation notes were written by surgical residents; this is concerning because previous research has shown that trainees struggle to produce high-quality operation notes without assistance^27^. According to the Pakistan study, the majority (86.5%) of operation notes were also written by trainee surgeons^24^. While globally, only 10–18% of institutions offer operative note writing as part of their residency program curricula, and most senior surgeons have never received such training, it has been shown that teaching operative note writing has improved the quality of documentation^18,28^.

Although all of the operation notes were assessed as legible and handwritten in this study, it has been shown that handwritten operation notes are often illegible^29^, while typed electronic notes have demonstrated full legibility^30,31^. For this reason, RCSE guidelines recommend that operation notes be typed whenever possible.

RCSE guidelines also call for the documentation of anticipated blood loss, antibiotic prophylaxis, and a total used instrument count, all of which were not regularly recorded in the operation notes that we analyzed. These omissions are particularly troubling, as a lack of properly documented instrument counts and antibiotic prophylaxis increases the likelihood of adverse safety incidents^32^, and a lack of blood loss estimation creates obstacles for adequate postoperative transfusion care if needed.

These omissions call for the systematization of both the body of the operation note form and of postoperative care orders linked to the operative findings. Incorporating these elements into a new format for operation notes, again linking to a preoperative safety checklist if possible, would align our operation notes with the RCSE’s 2014 operation note guidelines.

### Limitation

As a limitation, this study was conducted in an observational study design, and this may limit nationwide operation note guideline development.

## Conclusions

The practice of manual operation note documentation was below the standards recommended by the 2014 RCSE guidelines. Completion and documentation of surgical procedures in the study area operation sheets were excellent in terms of recording date, patient’s identity, surgeon, intraoperative finding documentation, procedure name, and postoperative plan. However, improvement is needed in documenting the urgency of the procedure, the occurrence or not occurrence of complications, the used instrument count, whether or not any extra procedures were conducted, and the estimated blood loss.

### Recommendations

We suggest that hospital administrators should use the RCSE guidelines as a baseline to standardize operation note-taking, with some modifications as needed following the guidelines, and should implement improved, standardized operation notes with clear, itemized sections for the preoperative state of the urgency of the procedure, team members, the date and time of surgery, intraoperative procedure details, and postoperative care instructions, as outlined above.

This amended format should include spaces to enter details, such as time, the names of all surgical team members, and blood loss estimations, which were commonly missing from the templates used during the period under study.

## Ethics approval and consent to participate

Ethical approval for this study (Ref No. 632) was provided by the Ethical Review Committee of the University of Gondar, College of Medicine and Health Science, School of Medicine, North Gondar, Ethiopia, on 20 March 2022.

## Consent

Written informed consent was obtained from participants after a clear explanation about the merits of the study for publication and any accompanying images. A copy of the written consent is available for review by the Editor-in-Chief of this journal on request.

## Sources of funding

The University of Gondar.

## Author contribution

This work was carried out in collaboration among all authors. N.G.T.: contributed to the conception, the review, and interpreted the result; D.Y.F., B.A.T., and B.M.A.: were involved in this title selection, commenting, editing the manuscript, assisting with data analysis, interpreting the result, and sending it for publication in your journal.

## Conflicts of interest disclosure

The authors declared that they have no conflicts of interest.

## Research registration unique identifying number (UIN)

1. Name of the registry: research registry.
2. Unique identifying number or registration ID: 8797.
3. Hyperlink to your specific registration (must be publicly accessible and will be checked): https://www.researchregistry.com/browse-the-registry#home

## Guarantor

Belete Muluadam Admassie, e-mail: uogbelete@gmail.com, Tel: +251 945567123, P.O. Box: 196.

## Data availability statement

All data generated or analyzed during this study were included in this published article and available on request.

## Provenance and peer review

Not commissioned, externally peer-reviewed.
